# Psychological Well-Being, Substance Use, and Internet Consumption Among Students and Teaching Staff of the Faculty of Veterinary Medicine: Risk and Protective Factors Associated with Well-Being and Dissatisfaction

**DOI:** 10.3390/healthcare13080918

**Published:** 2025-04-16

**Authors:** Irina Hernández-Trujillo, Elisa Hernández-Álvarez, Jaime Rojas-Hernández, Lucas F. Borkel, Tobias Fernández-Borkel, Domingo J. Quintana-Hernández, Luis Alberto Henríquez-Hernández

**Affiliations:** 1 Faculty of Veterinary, Universidad de Las Palmas de Gran Canaria, 35413 Arucas, Spain; irina.hernandez104@alu.ulpgc.es (I.H.-T.); elisa.hernandez106@alu.ulpgc.es (E.H.-Á.); 2 Asociación Científica Psicodélica, 35300 Canary Islands, Spain; jaime.rojas102@alu.ulpgc.es (J.R.-H.); lucas.fernandez101@alu.ulpgc.es (L.F.B.); tobiasfernandezborkel@gmail.com (T.F.-B.); domingoj.quintana@gmail.com (D.J.Q.-H.); 3 Asociación Canaria para el Desarrollo de la Salud a Través de la Atención, 35007 Las Palmas de Gran Canaria, Spain; 4 Health Sciences Faculty, Universidad de Las Palmas de Gran Canaria, 35016 Las Palmas de Gran Canaria, Spain; 5 Center for MR Research, University Children’s Hospital Zurich, 8032 Zurich, Switzerland; 6 Department of Forensic and Neurodevelopmental Sciences, Institute of Psychiatry, Psychology & Neuroscience, Kings College, London SE5 8AF, UK; 7 Faculty of Psychology, Universidad del Atlántico Medio, 35017 Las Palmas de Gran Canaria, Spain; 8 Unit of Toxicology, Clinical Science Department, Universidad de Las Palmas de Gran Canaria, 35016 Las Palmas de Gran Canaria, Spain

**Keywords:** psychological well-being, dissatisfaction, drug abuse, addictive behaviors, internet abuse, veterinary medicine

## Abstract

**Background:** Veterinary students experience high levels of mental health issues. **Objectives**: To analyze substance use, internet consumption, and mental health factors among students and academic staff of the Faculty of Veterinary Medicine of the University of Las Palmas de Gran Canaria, identifying factors associated with well-being and dissatisfaction. **Methods**: A total of 226 respondents participated, including 177 students (78.3%) and 49 staff members (21.7%). Data were collected between 30 October 2024 and 14 January 2025 using an adapted EDADES-based survey assessing substance use (alcohol, tobacco, electronic nicotine delivery systems (ENDSs), anxiolytics, and illicit drugs), internet habits, and psychological well-being among participants. Binary logistic regression was applied to identify factors associated with dissatisfaction. **Results**: Students exhibited higher binge drinking rates, greater ENDS consumption, and more problematic internet use than staff. Significant gender differences were observed, with females reporting greater emotional distress and a higher need for psychological support. Water pipe use (OR = 2.79, 95% CI = 1.45–5.38), anxiolytic consumption (OR = 2.31, 95% CI = 1.08–4.92), and excessive internet use (OR = 4.83, 95% CI = 1.66–14.1) were associated with lower overall satisfaction. Age was inversely associated with dissatisfaction (OR = 0.96, 95% CI = 0.94–0.98), and females were significantly more likely to report dissatisfaction (OR = 2.79, 95% CI = 1.45–5.38). **Conclusions**: Veterinary students exhibited higher psychological distress than teaching staff. Implementing targeted interventions to address substance use and internet habits is needed in order to enhance psychological well-being.

## 1. Introduction

According to the World Health Organization (WHO), health is a state of complete physical, mental, and social well-being, and not merely the absence of disease or infirmity [1]. Specifically, mental health is a state of mental well-being that enables people to cope with the stresses of life, realize their abilities, learn well and work well, and contribute to their community [2]. According to recent studies, the prevalence of mental disorders has risen from 2007–2009 to 2019–2022, with a more pronounced increase among students, young adults, and urban populations [3]. The COVID-19 pandemic has resulted in widespread mental health challenges, significantly affecting anxiety, depression, and stress levels across various demographics [4]. Between 2019 and 2022, high rates of anxiety and depression were identified among children and adolescents [5]. The effects on mental health persist to this day, with consequences observed across different populations [6]. In the context of higher education, the prevalence of depression in university undergraduates is 25%, and the prevalence of suicide-related outcomes is 14% [7].

Veterinary medicine presents unique mental health challenges due to heavy workloads, financial stress, and emotional burden, increasing mental health risks [8,9]. Veterinary students have been the focus of research due to their distinct characteristics: a strong vocational commitment and high sensitivity to animal welfare [10]. Veterinary students face challenges beyond companion animal care, such as livestock slaughter and malpractice, which contrast with their idealized perceptions of the profession. As a consequence, they report high levels of depression, anxiety, and suicidal thoughts [11]. Compared to the general population, veterinary students exhibit poorer overall well-being and higher levels of mental distress. Interestingly, their well-being did not significantly differ from that reported within the veterinary profession [12]. Therefore, rather than improving after graduation, mental health issues developed during studies persist into professional life, particularly in the form of burnout syndrome, with a higher prevalence among women with less professional experience [13]. Veterinary students face higher anxiety and depression levels, with gender, grade point average, and loneliness as key risk factors [14,15], particularly affecting female students.

The global prevalence of mental disorders has been estimated at 13.0%, with anxiety disorders being the most common (4.1%), followed by depressive disorders (3.8%), including major depressive disorder (2.5%) and dysthymia (1.3%) [16]. Among these disorders, depression stands out, not only due to its prevalence but also because of its impact on personal dissatisfaction, interpersonal relationships, and suicide risk [17,18]. As a response to depressive and isolating states, individuals tend to develop harmful habits that provide a temporary escape from their condition. The global prevalence of substance use disorders has been estimated at 2.2%, with alcohol use disorders (1.5%) being more common than other drug-related disorders (0.8%) [16]. Other substances considered legal, such as energy drinks, have been shown to be associated with a deterioration in mental health, especially among youth populations [19]. Psychological well-being and substance abuse have a complex, bidirectional relationship. Psychological traits, stressors, and early experiences influence vulnerability to drug use, while substance abuse can worsen mental health, triggering or exacerbating disorders like depression and anxiety [20,21]. Beyond its physiological effects, substance abuse often serves as a maladaptive coping mechanism for personal distress or social difficulties, highlighting its role as both a consequence and a contributor to psychological and social maladjustment [22]. Therefore, understanding drug abuse requires a holistic perspective that considers underlying mental health conditions, socio-environmental factors, and the reciprocal influences between substance use and emotional well-being.

Addictions are not limited to substance abuse. Internet addiction has emerged as a potential behavioral addiction, particularly affecting younger populations. The prevalence of smartphone addiction is 26.9% followed by social media addiction (17.4%) and internet addiction (14.2%) [23]. Internet addiction is influenced by a complex interplay of factors, including gender, age, and socioeconomic conditions [23]. Among university students, global prevalence of internet addiction is 41.8% [24]. Students in health fields are more vulnerable to internet addiction, which affects academic performance, sleep, and quality of life, and increases the risk of depression and suicide [25,26]. For decades, a significant positive association has been found between internet addiction and depression [27], with some studies suggesting that depression may contribute to internet addiction [28], while others propose that internet addiction may trigger depressive symptoms [29]. Although the direction of the association remains unclear, gender appears to be a significant risk factor associated with internet abuse. Thus, while males are more likely to engage in excessive internet use as a coping mechanism for depression, females are more prone to develop depression as a consequence of internet addiction [30].

The present study addresses a critical gap in the literature by focusing on the mental health challenges and substance use patterns in veterinary students and veterinary faculty staff, a population that has been largely overlooked despite growing evidence of occupational stress and burnout [13]. While previous studies focus on healthcare professionals, the mental health risks for veterinarians remain underexplored. Given the profession’s high-risk status, particularly among women, we hypothesize that veterinary students’ mental well-being is influenced by specific addictive behaviors, differing from those of faculty members. Thus, this study aimed to explore mental health and psychological well-being among students and teaching staff of the Faculty of Veterinary Medicine of the University of Las Palmas de Gran Canaria (Spain). Additionally, it examined the impact of substance use and internet behaviors on mental health, considering sociodemographic variables, differences in prevalence, consumption patterns, initiation age, and compulsive usage patterns. While external and personal factors may influence mental well-being, we focused on addictive behaviors in veterinary students and teaching staff. Future studies using longitudinal designs or controlled comparisons could help further differentiate the influence of profession-specific stressors from other individual or environmental factors.

## 2. Materials and Methods

### 2.1. Instrument

To assess the addictive behaviors of the study population, a shortened version of the EDADES survey on alcohol and drugs in Spain [31] was used (available at: https://pnsd.sanidad.gob.es/profesionales/sistemasInformacion/sistemaInformacion/pdf/2024_Informe_EDADES.pdf, accessed on 12 February 2025). This survey is published biennially by the Ministry of Health of the Government of Spain and is managed by the Government Delegation for the National Drug Plan (DGPNSD). The study primarily focuses on calculating the prevalence of substance use using four temporal indicators: lifetime, last 12 months, last 30 days prior to the survey, and daily use in the last 30 days. For alcoholic beverages, consumption indicators are estimated, including the prevalence of alcohol intoxication and consumption intensity. The survey includes the age of initiation for certain substances of abuse, the Compulsive Internet Use Scale (CIUS) [32], and questions related to mental health.

For the present study, a total of 67 questions were selected, divided into six sections, as follows: (1) alcoholic and energy drinks (15 questions); (2) tobacco, water pipes, and electronic nicotine delivery systems (ENDSs) (10 questions); (3) anxiolytics, sedatives, and/or hypnotics (5 questions); (4) drugs of abuse (11 questions); (5) internet use habits (15 questions); and (6) mental health and psychological well-being (11 questions). Age, gender, academic year (students only), and whether they were enrolled in subjects from previous academic years were requested. The final version of the questionnaire was developed using the Google Surveys platform. To ensure anonymity, the collection of IP addresses, cookies, and any other information that could reveal the identity of participants was disabled.

The questionnaire included an informative paragraph explaining the study’s objectives, anonymity, and confidentiality of the data. Participation was entirely voluntary, with no incentives or penalties associated with participation or non-participation. Informed consent was obtained by requiring participants to confirm their willingness to participate before proceeding with the survey. Participants could withdraw at any point without providing justification.

The questionnaire was launched on 30 October 2024, and remained open until 14 January 2025.

### 2.2. Study Population

The Faculty of Veterinary Medicine at the public University of Las Palmas de Gran Canaria is located on the Bañaderos campus (municipality of Arucas), approximately 20 km northwest of the capital of Gran Canaria (Canary Islands, Spain). The faculty is primarily composed of local students, as well as students from other islands of the archipelago. Exchange programs allow for the admission of three to five international students per year, starting from the second year of study. The teaching staff is predominantly of Spanish origin, mostly local. According to official data from the Dean’s Administration of the faculty, during the academic year 2024/2025, a total of 371 students were enrolled at the Faculty of Veterinary Medicine: 71 were enrolled in the first year, 77 in the second year, 66 in the third year, 57 in the fourth year, and 100 in the fifth year of study. In addition, the teaching and research staff was composed of 112 subjects. Participants were selected using a convenience sampling method. An official invitation to participate was sent via electronic communication from the Dean’s Office, ensuring a formal and structured distribution process. Additionally, the survey link was shared through social media platforms to maximize reach within the target population.

The study was approved by the Research Ethics Committee of the province of Las Palmas, Spain (ethical approval code #2024-449-1). All procedures were conducted in accordance with ethical guidelines for research involving human participants, including the principles outlined in the Declaration of Helsinki.

### 2.3. Data Collection and Statistical Analysis

The collected data were stored using Microsoft Excel (Microsoft Corporation, Redmond, WA, USA). The data collected were handled solely by the authors of this study. The data were only used for the purpose of this study, and there was no further procedure attempting to correlate the responses received with any specific responder.

Descriptive analyses were conducted for all variables. Means and standard deviations, medians and ranges, and the 25th and 75th percentiles of the distribution were calculated for continuous variables. Proportions were calculated for categorical variables. The normality of the data was tested using the Kolmogorov–Smirnov test. A continuous internet use scale was created based on responses to the 15 internet-related questions. The sum of the items resulted in a scale ranging from 15 to 75 points. A cut-off for “high internet use” was established at the 75th percentile of the distribution. The dataset was then dichotomized based on this cut-off, and subsequent statistical analyses were conducted. The same strategy was used for the variable “overall satisfaction”: it was categorized using the 75th percentile of the distribution. Dissatisfaction was considered when overall satisfaction was lower than the 75th percentile of the distribution. The dichotomized variable was used for subsequent analyses. Comparisons between groups were performed using parametric (Student’s *t*-test) or non-parametric tests (Mann–Whitney U test). Differences in the categorical variables were tested by the Chi-square test or Fisher’s exact test. Bivariate correlations between continuous variables were tested using Pearson’s r or Spearman’s rho, depending on the normality distribution of the variables. Binary logistic regression was used to predict the probability of an event occurring (odds ratio (OR)). In addition, multivariate analysis was performed including only those variables that were found to be significant in the univariate models. This approach was chosen to avoid overfitting and ensure that the model accurately reflected the most relevant predictors of the outcomes under study. Probability levels of <0.05 (two-tailed) were considered statistically significant. PASW Statistics (version 19.0, SPSS Inc., Chicago, IL, USA) was used to perform statistical analyses.

## 3. Results

The survey was distributed to 483 individuals, including students and teaching staff, of whom 226 (46.8%) completed it in full, with no missing data recorded. Among the respondents, 177 (78.3%) were students and 49 (21.7%) were teaching staff. According to official enrollment data for the 2024–2025 academic year, 47.7% of students and 43.7% of staff participated in the study. A total of 40 out of 57 (70.2%) and 65 out of 100 (65.0%) of fourth- and fifth-year students responded the questionnaire, respectively. Responders of first-, second-, and third-year students were 36.7%, 26.0%, and 39.4% from the total number of students enrolled in those courses, respectively.

Mean age among students was 22.5 ± 4.1 years, while the mean age among the staff was 47.8 ± 11.4 years (*p* < 0.001; Table 1). The majority of students were female (*n* = 143, 80.8%), whereas among the staff, most were male (*n* = 30, 61.2%). This difference in gender distribution was statistically significant (*p* < 0.001), indicating a disparity between the two groups. A total of 81 students (45.8%) had subjects from previous years.

### 3.1. Consumption of Alcohol and Energy Drinks

Table 2 presents the descriptive analysis of alcohol and energy drink consumption. A total of 214 respondents (94.7%) reported alcohol use, with 54.0% (122/226) consuming it in the last 30 days. The mean age at which participants reported getting drunk for the first time was 17.1 ± 2.6 years, though 26.1% had never been drunk. Most drank once a month or less (64.2%), consuming 1–2 drinks (66.8%). Most responders had not felt guilty (76.6%), had not blackouts (86.0%), had not experienced accidents (96.7%), or had not been warned to stop drinking (95.8%).

Significant differences in alcohol consumption patterns were observed between students and staff. A total of 73.5% of staff consumed alcohol in the last 30 days, compared to 48.6% of students (Q1; *p* = 0.006). However, 18.6% of students reported intoxication in the same period, versus 4.1% of staff (Q2; *p* < 0.001). Students also had a lower age of first intoxication (Q3; 16.6 ± 1.8 vs. 18.6 ± 3.8 years, *p* < 0.001) and a lower drinking frequency (Q6; *p* < 0.001). A total of 28.7% of students participated in “botellón”, defined as social gatherings, typically among young people, where large quantities of alcoholic beverages are consumed in public spaces without formal regulation or supervision, compared to 2.0% of staff (Q4; *p* < 0.001). Motivations differed, with students drinking for fun (41.9%), while staff drank for the sensation (55.3%) (Q5; *p* < 0.001).

Regarding energy drinks, 36.7% of respondents consumed them in the last 30 days (Q12), with higher intake among students (40.7% vs. 22.4%, *p* = 0.020). Most consumed them once a month or less (Q13). Alcohol–energy drink combinations (Q14) were more common in students (37.5% vs. 9.1%), though not statistically significant. Both groups consumed this combination infrequently (Q15).

### 3.2. Consumption of Tobacco, Water Pipes, and Electronic Nicotine Delivery Systems (ENDSs)

In the whole series, 46.5% of respondents had never smoked (Q16). Among smokers, the mean initiation age was 18.8 ± 4.8 years (range: 14–42, Q19), with a monthly average of 105.5 cigarettes (Q17), primarily hand-rolled (44.1%, Q18). A total of 65.6% had unsuccessfully attempted to quit (Q20). No significant differences were found between students and staff (Table S1).

For water pipe use (Q21), consumption among students was 7.3% in the past year and 3.4% in the last 30 days, while staff reported 0% (*p* = 0.042). Regarding ENDS use (Q23), prevalence was significantly higher among students (37.3%) than staff (10.2%). Students reported higher consumption rates in the past year (7.3% vs. 0%), last 30 days (8.5% vs. 4.1%), and daily use (1.1% vs. 0%; *p* < 0.001). Initiation age was significantly lower for students (23.3 ± 7.1 years vs. 44.0 ± 5.6 years, *p* < 0.001, Q24). While 12.5% of students used nicotine-free cartridges, 57.1% of staff preferred nicotine-containing ones (Q25; *p* = 0.014, Table S1).

Bivariate logistic regression showed that age was a variable associated with increased odds for tobacco consumption (OR = 1.03, 95% CI = 1.01–1.06; *p* = 0.009, Table S2), but it was inversely associated with ENDS consumption (OR = 0.95, 95% CI = 0.92–0.98; *p* < 0.001). Additionally, male staff members had a higher risk of water pipe use (OR = 6.09, 95% CI = 1.46–25.4; *p* = 0.013).

### 3.3. Consumption of Anxiolytics, Sedatives, and/or Hypnotics

In the whole series, 23.0% of respondents had used anxiolytics, sedatives, and/or hypnotics sometime in life (Q26), with 86.3% initiating use over a year ago (Q28). Most obtained them through personal prescriptions (63.0%), while 26.0% used prescriptions for others and 11.0% acquired them without prescriptions (Q29). A total of 11.7% combined them with alcohol or illicit drugs, though 96.3% used them alone (Q30) (Table S3).

Initiation age was significantly lower among students (19.6 ± 3.2 years) than staff (33.9 ± 10.5 years, *p* < 0.001, Q27), indicating different consumption patterns between both groups (Table S3). Bivariate logistic regression showed that age was a factor linked to anxiolytic consumption (OR = 1.03, 95% CI = 1.01–1.05; *p* = 0.012, Table S2).

### 3.4. Consumption of Drugs of Abuse

The descriptive analysis of drug abuse consumption in contained in Table S4. The majority of respondents (54.9%) reported never using cannabis, marijuana, or hashish, while 34.1% had consumed it at least once in life (Q31). The mean initiation age was 18.4 ± 3.2 years, with no differences between students and staff. However, males had a higher prevalence than females (54.7% vs. 41.4%, *p* = 0.048). Most cannabis users (94.6%) had last consumed it over a year ago (Q33). Regarding cocaine (Q34), staff had a higher proportion of consumption than students (20.4% vs. 4.5%, *p* < 0.001), with males reporting more frequent use than females (14.1% vs. 5.6%, *p* = 0.036). 3,4-Methylenedioxymethamphetamine (MDMA; Q36) and volatile inhalant (Q40) use sometime in life were low (4.0% and 5.8%), but 11.1% and 21.7% of users had consumed them in the past year, the highest rate among all substances (Q37, Q41). MDMA use was higher among males (17.2% vs. 4.3%, *p* = 0.004). Amphetamine use was more common among staff than students (18.4% vs. 3.4%, *p* = 0.001; Table S4) and significantly higher in males (15.6% vs. 4.3%, *p* = 0.009).

Additionally, bivariate logistic regression showed that age was a factor linked to illicit drug use (OR = 1.03, 95% CI = 1.01–1.05; *p* = 0.012, Table S2).

### 3.5. Internet Use Behaviors and Potentially Associated Factors

Significant differences were observed between students and staff regarding internet use patterns, with students exhibiting greater compulsive and problematic usage. A total of 7.9% of students reported never struggling to stop using the internet, compared to 40.8% of staff (*p* < 0.001, Q42). Similarly, only 12.4% of students reported never continuing internet use despite wanting to stop, while 53.1% of staff had never experienced this issue (*p* < 0.001, Q43). Students were more frequently advised by others to reduce internet use (*p* = 0.008, Q44) and were significantly more likely to prioritize online activities over social interactions (*p* < 0.001, Q45). Additionally, 8.5% of students frequently prioritized online activities, while this behavior was absent among staff. Regarding the impact on daily functioning, students were far more likely to experience sleep deprivation due to internet use. Only 17.5% of students had never lost sleep from being online, compared to 57.1% of staff (*p* < 0.001, Q46). Similarly, 20.3% of students reported frequent sleep loss, versus 2.0% of staff (Table 3). Students were more prone to neglect responsibilities due to internet use (Q51, Q52), with only 35.6% of students stating they had never done so, compared to 75.5% of staff (Q52; *p* < 0.001). A total of 24.4% of students admitted to prioritizing internet use sometimes, often, or very frequently, while none of the staff reported this behavior (*p* < 0.001, Q51). Students also had greater difficulty limiting their usage, with 14.7% often failing in their attempts, compared to 4.1% of staff (*p* < 0.001, Q50).

Students more frequently experienced urges to go online (Q48) and persistent thoughts about internet use even when offline (Q47). Only 37.9% of students had never experienced these thoughts, compared to 67.3% of staff (*p* < 0.001, Q47). Similarly, 53.1% of staff had never felt the urge to go online, compared to 23.2% of students (*p* < 0.001, Q48). Students were also significantly more likely to use the internet as a coping mechanism for stress or negative emotions (29.9% vs. 4.1%, *p* < 0.001, Q54) and to go online often when feeling down (33.3% vs. 6.1%, *p* < 0.001, Q53). A total of 40.7% of students never felt anxious or irritable when unable to go online, compared to 73.5% of staff (*p* = 0.001, Q55). However, students appeared more aware of their problematic internet use: 17.5% frequently thought they should use it less, while no staff members reported this concern (*p* < 0.001, Q49). Despite these differences, most respondents (77.0%) had never experienced online mistreatment, with no significant differences between groups (Q56; Table 3).

**Figure 1:**
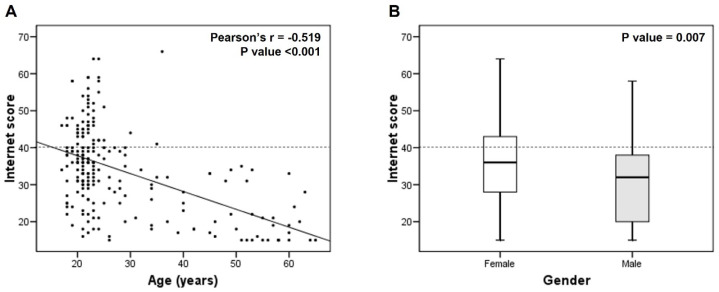
Bivariate correlation between age and internet use score (**A**) and box plot representing the difference in internet use score by gender (**B**). The line represents the median, the box edges represent the 25th and 75th percentiles of the distribution, and the whiskers represent the range. Student’s *t*-test was employed to compare the means of the two groups (35.2 ± 11.2 vs. 30.7 ± 11.4, for females and males, respectively). The dashed line marks the 75th percentile of the internet use score. Prepared by the authors.

A correlation between age and high internet use was found, indicating that younger respondents had higher scores (Pearson’s r = −0.519, *p* < 0.001; Figure 1A). Notably, no staff members reported an internet score above the 75th percentile (40 points). Regarding gender differences, females had significantly higher internet scores than males (35.2 ± 11.2 vs. 30.7 ± 11.4, *p* = 0.007; Figure 1B).

Factors linked to excessive internet use (≥40 points) were analyzed (Table S2). Females (OR = 2.42, 95% CI = 1.11–5.30; *p* = 0.027), energy drink consumption (OR = 2.19, 95% CI = 1.18–4.06; *p* = 0.013), water pipe use (OR = 3.11, 95% CI = 1.60–6.04; *p* = 0.001), and ENDS consumption (OR = 2.65, 95% CI = 1.41–4.97; *p* = 0.002) were variables associated with increased odds for high internet use. In contrast, age was a variable associated with decreased odds for excessive internet use (OR = 0.91, 95% CI = 0.85–0.96; *p* = 0.001). When all significant variables were included in the model, age (OR = 0.88, 95% CI = 0.81–0.96; *p* = 0.005) and water pipe use (OR = 3.78, 95% CI = 1.50–9.60; *p* = 0.005) remained significant predictors of excessive internet use.

### 3.6. Mental Health, Psychological Well-Being, and Potentially Associated Factors

A total of 41.2% of students had considered taking medical leave for emotional reasons in the past year, compared to 14.3% of staff (*p* < 0.001, Q57, Table 4). Students were more likely to be diagnosed with anxiety or depression (22.0% vs. 8.2%, *p* = 0.038, Q58), and 42.4% suspected having these conditions without a diagnosis, compared to 10.2% of staff (*p* < 0.001, Q59). Suicidal ideation (Q60) was higher among students (13.0%) than staff (2.0%), though not statistically significant (*p* = 0.066). However, suicide planning (Q61) was significantly more common in students (11.9% vs. 2.0%, *p* = 0.034). Three students reported a suicide attempt in the past year (Q62). Additionally, 19.2% of students lacked a safe space for emotional expression, compared to 4.1% of staff (*p* < 0.001, Q63). Students were significantly more likely to seek professional help (28.8% vs. 8.2%, *p* < 0.001, Q64), yet staff were more likely to state they did not need help (63.3%), suggesting stronger self-regulation mechanisms. Regarding life satisfaction (Q66, Q67), staff reported higher overall satisfaction than students (61.2% vs. 46.3%, *p* = 0.022), with a significantly higher median satisfaction score (83 vs. 75, *p* < 0.001).

We also explored the influence of gender in relation to questions concerning mental health and psychological well-being (Table S5). Females (39.5%) were more likely than males (25.0%, *p* = 0.040, Q57) to consider interrupting studies for emotional reasons. They were also more likely to suspect they had anxiety or depression (40.7% vs. 21.9%, *p* < 0.001, Q59). Females were less likely to have a safe emotional space (17.3% vs. 12.5%, *p* = 0.018, Q63), yet more likely to report having such a space available (50.0% vs. 34.4%), suggesting a contradiction between emotional expression and perceived support. Additionally, 27.2% of females sought professional help but had not accessed it, compared to 17.2% of males (*p* = 0.001, Q64), while males were more likely to believe they did not need help (53.1% vs. 25.3%), indicating greater self-reliance or lower mental health awareness. Finally, males reported higher life satisfaction (median = 80 vs. 75, *p* = 0.001, Q67).

**Figure 2:**
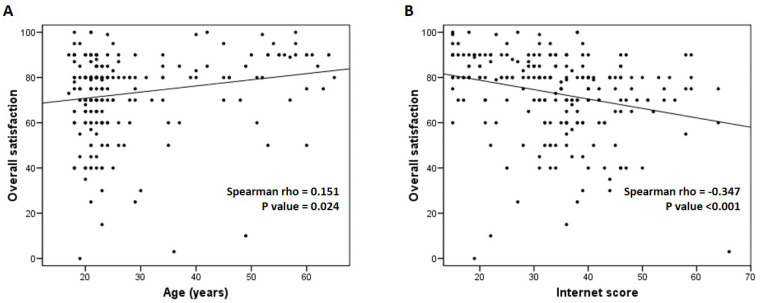
Bivariate correlation between age and overall satisfaction (ranged 0–100) (**A**) and between internet score (ranged 15–75) and overall satisfaction (**B**). Prepared by the authors.

A correlation between age and overall satisfaction was found (Spearman’s rho = 0.151, *p* = 0.024; Figure 2A). Additionally, we observed a correlation between internet use and overall satisfaction (Spearman’s rho = −0.347, *p* < 0.001; Figure 2B). Significant differences in overall satisfaction were observed based on gender (Figure S1A), water pipe use (Figure S1B), anxiolytic consumption (Figure S1C), and excessive internet use (Figure S1D). Females (median = 75 vs. 80, *p* = 0.001), water pipe users (median = 75 vs. 80, *p* = 0.013), anxiolytic consumers (median = 70 vs. 80, *p* = 0.001), and individuals with high internet use (median = 70 vs. 80, *p* = 0.001) reported significantly lower satisfaction levels.

Age was inversely associated with dissatisfaction, with older individuals less likely to report it (OR = 0.96, 95% CI = 0.94–0.98; *p* < 0.001, Table 5). Variables associated with increased odds included female gender (OR = 2.40, 95% CI = 1.25–4.62; *p* = 0.009), water pipe use (OR = 2.79, 95% CI = 1.45–5.38; *p* = 0.002), and anxiolytic consumption (OR = 2.31, 95% CI = 1.08–4.92; *p* = 0.031). Among students, enrolling in subjects from previous years was also associated with dissatisfaction (OR = 2.52 (1.10–5.82); *p* = 0.030). Internet use, as both a continuous and dichotomized variable, was linked to dissatisfaction (OR = 1.08, 95% CI = 1.04–1.11; *p* < 0.001; OR = 4.83, 95% CI = 1.66–14.1; *p* = 0.004). When all significant variables were included in the model, water pipe use (OR = 2.10, *p* = 0.046), anxiolytic consumption (OR = 2.83, *p* = 0.018), and internet use (OR = 1.09, *p* = 0.007) remained significant predictors.

## 4. Discussion

The present study examines the pattern of use of various psychoactive substances, both legal and illegal, internet usage patterns, and mental health status at the Faculty of Veterinary Medicine of the University of Las Palmas de Gran Canaria. Although this study targets veterinary students and staff, broader sociodemographic factors—such as low income, limited education, and adverse family settings—also contribute significantly to substance use and mental health outcomes rather than exclusively by their academic or professional context [33,34]. Socioeconomic status, access to healthcare, and social environments also influence these behaviors in the general population [35]. As our study lacked detailed socioeconomic data, future research should explore these factors to better understand their influence on the observed associations.

### 4.1. Patterns of Legal and Illegal Substance Use

The latest edition of the EDADES survey, published by the Ministry of Health of the Government of Spain, dates to 2024 [31] (available at: https://pnsd.sanidad.gob.es/profesionales/sistemasInformacion/sistemaInformacion/pdf/2024_Informe_EDADES.pdf, accessed on 12 February 2025). It is important to note that the EDADES survey targets the general population, whereas the present study focuses on a specific population, meaning that observed differences may be attributed to the inherent characteristics of our sample.

Overall, men exhibit higher prevalence rates for all psychoactive substances, except for hypnosedatives and opioid analgesics (not considered in the present study), which are more frequently consumed by women [31]. In our study, men reported higher consumption of THC, cocaine, MDMA, and amphetamines, but no significant differences were observed regarding hypnosedatives. In any case, lifetime consumption of these medications was 23.0%, a prevalence similar to that of the general population (27.4%) [31]. Furthermore, our findings align with official data in identifying age as a predictor of their consumption. Our results indicate that, in the whole series, the lifetime prevalence of THC, cocaine, MDMA, and amphetamines consumption was 34.1%, 6.6%, 4.0%, and 6.6%, respectively. In the general population, these prevalence rates were 43.7%, 13.0%, 5.1%, and 4.5%, which are higher than those reported in our study population [31]. However, MDMA consumption in the last 12 months was 0.7% in the general population, whereas it reached 6.1% among faculty members and 2.3% among veterinary students. Similarly, the prevalence of volatile inhalants was higher in our cohort (5.8%) compared to the general population (<1.0%) [31]. Previous studies have shown that MDMA consumption prevalence is higher among university populations. Compared to other universities, the prevalence observed in our study was lower than that reported among students at the University of Girona (11.1%) or Paris (21.5%) [36,37], and similar to that reported in other institutions [38,39]. This increase in MDMA consumption among university students has been observed since the 1990s [40]. A similar pattern is observed with poppers, the main volatile inhalant, which is widely used among university students [41]. Its consumption is often associated with alcohol use and an increased risk of unsafe sexual practices [42], mainly among males. However, we did not observe differences in the prevalence of consumption by gender.

According to the EDADES survey, the mean age of initiation in the general population is 18.4 years for cannabis, 16.6 for tobacco, and 16.4 for alcohol [31]. In the present study, participants reported a similar age of initiation for cannabis (18.4 years), but slightly later for tobacco (18.8 years) and alcohol (17.1 years). However, the age of alcohol initiation was significantly lower among students (16.6 years) compared to faculty members (18.6 years). In the year 2024, 92.9% of the population aged 15 to 64 years reported having consumed alcoholic beverages at some time in their lives, slightly more than 76% reported having drunk alcohol at some time during the last 12 months, and 63.5% reported having done so in the last 30 days [31]. These data are higher than those observed in the present study. However, the prevalence of acute alcohol intoxication in the last 12 months was 14.7% in the general population, being 20.4% in the present study and significantly higher among veterinary students (22.0%). The lower prevalence in frequency in contrast to the higher prevalence in intensity shows a specific characteristic of our population. This pattern of consumption has been previously observed in other studies conducted in higher education students. The percentage of students who have reported binge drinking is 13.0% in universities in Italy [43] and 20.0% in the United Kingdom and Ireland [44]. The reasons that contribute to understanding this compulsive behavior in relation to alcohol are varied, including pressure and social phobia or poor academic achievement [45]. The prevalence of energy drink consumption in the past 30 days in the population aged 15 to 64 years has risen since 2022, currently standing at 16.5%, exceeding 50% among men aged 15 to 24 years [31]. In the present series, we did not observe differences by sex, but we did observe a prevalence in the complete series of 36.7%, being significantly higher (40.7%) among students. Regarding tobacco and ENDSs, the prevalence of lifetime use in the general population is 66.6% and 19.0%, respectively [31], and was 35.4% and 31.4%, respectively, in the present study population. The data referring to ENDSs should be taken into account, since it has been observed that their consumption is associated with higher levels of depression, anxiety, and stress [46].

### 4.2. Internet-Related Behaviors and Psychological Well-Being

The official EDADES 2022 report indicates that 3.5% of the general population aged 15 to 64 years engage in problematic internet use [47]. To make this calculation, the 14 CIUS questions have a maximum total score of 56, so that those individuals with 28 points or more are considered to be in the group with an abusive use of the Internet. In the present study, the maximum score was 75 points, establishing the cut-off at the 75th percentile of the distribution, which corresponded to 40 points. Therefore, no real quantitative comparison can be made. However, even though the method used in the present study was more restrictive, the number of respondents above the cut-off point was 55, which represents 24.3% of the series. It should be noted that no member of the teaching staff was above this cut-off point. This compulsive behavior in relation to the Internet has been previously explored in the context of higher education, where healthcare students appear to be particularly at risk [24].

According to the EDADES 2024 survey, 2.2% of the population acknowledges having had suicidal thoughts, 1.6% have had suicide plans and 0.5% have attempted suicide. Additionally, individuals aged ≤34 are at higher risk for suicidal ideation or planning, with females being particularly vulnerable [31]. In the present study, suicidal ideation was present in 10.6% of the series, 9.7% have made suicide plans (a significantly higher percentage among students (11.9%)), and 1.1% reported having attempted suicide in the last year. This suggests that, as previously established, mental well-being is lower among veterinary students [11], highlighting the need for a more in-depth analysis of the factors affecting this specific population.

### 4.3. Behavioral Contributors to Mental Health and Dissatisfaction

In terms of overall dissatisfaction, age was inversely associated, consistent with findings from the EDADES survey [31]. This does not imply that age should be targeted directly in interventions but rather suggests that interventions should consider demographic factors, including age, when designing tailored strategies for addressing mental health in specific subgroups. Conversely, female gender was identified as a predictor of dissatisfaction, aligning with observations in the general population [31]. Additional factors associated with dissatisfaction included water pipe use, anxiolytic consumption, and internet abuse, as evidenced in both univariate and multivariate analyses. Several studies have shown an association between water pipe use and poorer mental health among adolescents, university students, and young adults [48,49,50]. Furthermore, problematic internet use, water pipe consumption, and depression appear to be interrelated, creating a complex interaction where it is difficult to distinguish clear cause-and-effect relationships [48]. Apart from these, there may be additional ones, such as energy drink consumption, water pipe use, and ENDS consumption, which have also been identified as factors linked to internet abuse in the present study. Moreover, energy drinks consumption has been associated with various health and well-being concerns, particularly in adolescents and young adults [19,51]. It should be highlighted that the veterinary profession exhibits higher suicide rates than the general population, placing this group at risk [52,53]. Therefore, implementing preventive measures is necessary from the university education stage.

The relationship between depression and problematic internet use has been recognized since the 1990s [27], linked to greater loneliness, depression, and reduced communication with family and friends, a phenomenon known as “the internet paradox” [54]. While the risk is higher among adolescents [55], higher education students, particularly those in health-related fields, are not exempt from this vulnerability [28]. The present study supports “the internet paradox”, showing that veterinary students share this vulnerability, with excessive internet use increasing distress.

Overall, the study emphasizes the need for a multimodal approach and support, as students are at high risk for mental illness and harmful behaviors. However, it is important to note that our study identifies associations rather than a direct causal relationship between these factors and the development of risky behaviors.

## 5. Strengths and Limitations

This study has several limitations that must be considered when interpreting the results. Firstly, although a validated questionnaire was used, socially desirable answers must be considered a potential bias. This is particularly relevant in the older population group, due to the stigma typically associated with mental health. To reduce social desirability bias, the anonymous online survey used neutral wording, and participants were assured of confidentiality. Secondly, a shortened version of the EDADES survey on alcohol and drugs in Spain was used to assess substance use. While this is a validated instrument at the national level, it has not been specifically validated for veterinary students and teaching staff. Although its widespread application supports its reliability, its applicability to this specific population has not been validated. The study design and the use of the EDADES survey only allow for comparisons with the data derived from that survey; therefore, direct comparisons with other studies cannot be conducted. Thirdly, several important factors that are known to influence mental health and well-being, such as childhood experiences, socioeconomic status, interpersonal relationships, social support, housing conditions, and past or current physical or mental health disorders, were not included in the validated questionnaire used. Moreover, the role of other substances and addictive behaviors (e.g., caffeine consumption in forms other than energy drinks) was not explored in the present study. Future research should consider quality of life to better understand factors influencing mental health in this population. Fourth, the results obtained in the present cohort should be compared with those of other healthcare-related students from the same institution, such as medical or nursing students. Substance use and mental health patterns may also reflect broader sociodemographic influences beyond the academic context.

Conversely, the present study offers several notable strengths. First, the study utilized the EDADES survey, a widely validated tool in Spain, which provides reliable baseline data for substance use and mental health patterns and allows for comparison within the Spanish context. While the demographic characteristics of the sample may not be directly comparable to the general population, they are representative of the specific population at the Faculty of Veterinary Medicine, where a higher proportion of students are women and the age range is generally younger. This demographic profile is consistent with the student and staff population of the Faculty, making the sample relevant and appropriate for the study’s objectives. Second, the study included both students and faculty members, with participation rates of 47.7% and 43.7%, respectively. This allowed for meaningful comparisons between these two key groups within the faculty, supporting a more comprehensive internal analysis. Third, the study benefits from the “pure” nature of the population, specifically focusing on veterinary students and staff from a single institution. This controlled population minimizes potential confounders from external factors, allowing for more accurate and meaningful interpretations of the results. Finally, the study achieved a 100% completion rate for the questionnaires, ensuring that all responses are fully accounted for and reducing the potential for response bias. This provides a high level of reliability and confidence in the data obtained.

## 6. Conclusions

Veterinary students showed higher psychological distress than teaching staff, including greater anxiety, depression, suicidal ideation, and need for support. Female students reported lower life satisfaction. Risk factors included younger age, female gender, water pipe and anxiolytic use, and high internet scores. Students exhibited more binge drinking, earlier initiation, and greater use of ENDSs and water pipes. MDMA and inhalant use were more common among students, and higher than consumption rates in the general population. Internet overuse was linked to sleep and social problems, especially in females. Energy drinks, water pipes, and ENDSs were associated with problematic use. Results mainly apply to veterinary students and should not be overgeneralized.

This study highlights the need for targeted interventions to address substance use and internet habits to improve psychological well-being in the studied population.

## Figures and Tables

**Table 1:** Population characteristics and descriptive analysis of the whole series and segmented by type of responder. The total number of responses is included, with the percentage in brackets.

		Type of Responder		
Variable	Whole Series (*n* = 226)	Students (*n* = 177)	Staff (*n* = 49)	*p* Value
Age (years)				
Mean ± SD ^a^	27.9 ± 12.3	22.5 ± 4.1	47.8 ± 11.4	<0.001 ^b^
Median (Range)	22 (17–65)	22 (17–45)	51 (26–65)	<0.001 ^c^
Gender				<0.001 ^d^
Female	162 (71.7)	143 (80.8)	19 (38.8)	
Male	64 (28.3)	34 (19.2)	30 (61.2)	
Type of responder				NA
Student	177 (78.3)	—	—	
Staff	49 (21.7)	—	—	
Year of study				NA
First	—	26 (14.7)	—	
Second	—	20 (11.3)	—	
Third	—	26 (14.7)	—	
Fourth	—	40 (22.6)	—	
Fifth	—	65 (36.7)	—	
Subjects from previous years *				NA
No	—	96 (54.2)	—	
Yes	—	81 (45.8)	—	

Abbreviations: SD, standard deviation; NA, not applicable. ^a^ Age followed a normal distribution only in the subgroup of teacher respondents. ^b^ Student’s *t*-test. ^c^ Mann–Whitney U-test. ^d^ Chi-square test. * Refers to students who, although enrolled in their current year, have not yet passed certain subjects from previous years in their academic program. Prepared by the authors.

**Table 2:** Descriptive analysis of the consumption of alcohol and energy drinks in the whole series and segmented by type of responder. The total number of responses is included, with the percentage in brackets.

		Type of Responder		
Question	Whole Series	Students	Staff	*p* Value ^#^
Q1. Have you consumed any type of alcoholic beverage?				0.006
Never	12 (5.3)	10 (5.6)	2 (4.1)	
Sometime in life	36 (15.9)	32 (18.1)	4 (8.2)	
In the last 12 months	55 (24.3)	49 (27.7)	6 (12.2)	
In the last 30 days	122 (54.0)	86 (48.6)	36 (73.5)	
Daily	1 (0.4)	0	1 (2.0)	
Q2. Have you ever been drunk?				<0.001
Never	59 (26.1)	53 (29.9)	6 (12.2)	
Sometime in life	85 (37.6)	51 (28.8)	34 (69.4)	
In the last 12 months	46 (20.4)	39 (22.0)	7 (14.3)	
In the last 30 days	35 (15.5)	33 (18.6)	2 (4.1)	
Daily	1 (0.4)	1 (0.6)	0	
Q3. At what age did you get drunk for the first time? (years) *				
Mean ± SD	17.1 ± 2.6	16.6 ± 1.8	18.6 ± 3.8	<0.001 ^a^
Median (Range)	17 (12–30)	16 (12–21)	18 (13–30)	0.002 ^b^
Q4. In the past 12 months, have you participated in public binge drinking (botellón)? **				<0.001
No	165 (77.1)	119 (71.3)	46 (93.9)	
Yes	49 (22.1)	48 (28.7)	1 (2.0)	
Q5. In the last 12 months, what was your main reason for drinking alcohol? **				<0.001
No consumption	39 (18.2)	33 (19.8)	6 (12.8)	
I like how I feel	78 (36.4)	52 (31.1)	26 (55.3)	
It is fun	78 (36.4)	70 (41.9)	8 (17.0)	
Just to get drunk	3 (1.4)	3 (1.8)	0	
To fit in my group	8 (3.7)	7 (4.2)	1 (2.1)	
Part of a healthy diet	8 (3.7)	2 (1.2)	6 (12.8)	
Q6. How often do you consume alcoholic beverages? **				<0.001
Once a month or less	115 (64.2)	100 (74.1)	15 (34.1)	
2–4 times a month	55 (30.7)	33 (24.4)	22 (50.0)	
2–3 times per week	8 (4.5)	2 (1.5)	6 (13.6)	
≥4 times per week	1 (0.6)	0	1 (2.3)	
Q7. How many alcoholic drinks do you typically consume when you drink? **				0.159
1–2	143 (66.8)	108 (64.7)	35 (74.5)	
3–4	51 (23.8)	41 (24.6)	10 (21.3)	
5–6	15 (7.0)	13 (7.8)	2 (4.3)	
7–9	4 (1.9)	4 (2.4)	0	
≥10	1 (0.5)	1 (0.6)	0	
Q8. How often, in the past year, have you felt guilty after drinking? **				0.395
Never	164 (76.6)	126 (75.4)	38 (80.9)	
<1 a month	45 (21.0)	36 (21.6)	9 (19.1)	
Monthly	4 (1.9)	4 (2.4)	0	
Weekly	1 (0.4)	1 (0.6)	0	
Daily	0	0	0	
Q9. How often, in the past year, have you been unable to remember what happened the night before because you had been drinking? **				0.882
Never	184 (86.0)	144 (86.2)	40 (85.1)	
<1 a month	27 (12.6)	20 (12.0)	7 (14.9)	
Monthly	1 (0.5)	1 (0.6)	0	
Weekly	0	0	0	
Daily	2 (0.9)	2 (1.2)	0	
Q10. Have you, or someone else, been injured as a result of your drinking? **				0.616
No	207 (96.7)	161 (96.4)	46 (97.9)	
Yes (but not this year)	6 (2.8)	5 (3.0)	1 (2.1)	
Yes (this year)	1 (0.5)	1 (0.6)	0	
Q11. Has anyone expressed concern about your alcohol consumption? **				0.966
No	205 (95.8)	160 (95.8)	45 (95.7)	
Yes (but not this year)	3 (1.4)	3 (1.8)	0	
Yes (this year)	6 (2.8)	4 (2.4)	2 (4.3)	
Q12. Have you consumed any energy drinks in the past 30 days?				0.020
No	143 (63.3)	105 (59.3)	38 (76.6)	
Yes	83 (36.7)	72 (40.7)	11 (22.4)	
Q13. How often do you consume energy drinks? ***				0.214
Once a month or less	52 (62.7)	42 (58.3)	10 (90.9)	
2–4 times a month	20 (24.1)	19 (26.4)	1 (9.1)	
2–3 times per week	7 (8.4)	7 (9.7)	0	
≥4 times per week	4 (4.8)	4 (5.6)	0	
Q14. Have you combined energy drinks and alcohol in the last 30 days? ***				0.089
No	55 (66.3)	45 (62.5)	10 (90.9)	
Yes	28 (33.7)	27 (37.5)	1 (9.1)	
Q15. How often do you consume energy drinks mixed with alcohol? ***				NA
Once a month or less	28 (100)	27 (100)	1 (100)	
2–4 times a month	0	0	0	
2–3 times per week	0	0	0	
≥4 times per week	0	0	0	

Abbreviation: NA, not applicable. ^#^ Chi-square test. * Only among responders who have been drunk. ** Only among responders who have consumed alcoholic beverages. *** Only among responders who have consumed energy drinks. ^a^ Student’s *t*-test. ^b^ Mann–Whitney U-test. Prepared by the authors.

**Table 3:** Descriptive analysis of the internet use habits in the whole series and segmented by type of responder. The total number of responses is included, with the percentage in brackets.

		Type of Responder		
Question	Whole Series	Student	Staff	*p* Value ^#^
Q42. How often have you found it difficult to stop using the internet once you started?				<0.001
Never	34 (15.0)	14 (7.9)	20 (40.8)	
Rarely	39 (17.3)	25 (14.1)	14 (28.6)	
Sometimes	85 (37.6)	74 (41.8)	11 (22.4)	
Often	49 (21.7)	45 (25.4)	4 (8.2)	
Very frequently	19 (8.4)	19 (10.7)	0	
Q43. How often have you continued using the internet even though you wanted to stop?				<0.001
Never	48 (21.2)	22 (12.4)	26 (53.1)	
Rarely	53 (23.5)	40 (22.6)	13 (26.5)	
Sometimes	71 (31.4)	62 (35.0)	9 (18.4)	
Often	38 (16.8)	37 (20.9)	1 (2.0)	
Very frequently	16 (7.1)	16 (9.0)	0	
Q44. How often do your parents or friends tell you that you should spend less time on the internet?				0.008
Never	84 (37.2)	55 (31.1)	29 (59.2)	
Rarely	74 (32.7)	65 (63.7)	9 (18.4)	
Sometimes	51 (22.6)	43 (24.3)	8 (16.3)	
Often	14 (6.2)	11 (6.2)	3 (6.1)	
Very frequently	3 (1.3)	3 (1.7)	0	
Q45. How frequently do you prioritize online activities over social interactions?				<0.001
Never	78 (34.5)	48 (27.1)	30 (61.2)	
Rarely	95 (42.0)	81 (45.8)	14 (28.6)	
Sometimes	37 (16.4)	32 (18.1)	5 (10.2)	
Often	15 (6.6)	15 (8.5)	0	
Very frequently	1 (0.4)	1 (0.6)	0	
Q46. How often do you get less sleep because of being online?				<0.001
Never	59 (26.1)	31 (17.5)	28 (57.1)	
Rarely	59 (26.1)	46 (26.0)	13 (26.5)	
Sometimes	62 (27.4)	55 (31.1)	7 (14.3)	
Often	37 (16.4)	63 (20.3)	1 (2.0)	
Very frequently	9 (4.0)	9 (5.1)	0	
Q47. How frequently do you have thoughts about being online, even when you’re offline?				<0.001
Never	100 (44.2)	67 (37.9)	33 (67.3)	
Rarely	82 (63.3)	67 (37.9)	15 (30.6)	
Sometimes	30 (13.3)	30 (16.9)	0	
Often	11 (4.9)	10 (5.6)	1 (2.0)	
Very frequently	3 (1.3)	3 (1.7)	0	
Q48. How often do you feel the urge to go online?				<0.001
Never	67 (29.6)	41 (23.2)	26 (53.1)	
Rarely	84 (37.2)	66 (37.3)	18 (36.7)	
Sometimes	52 (23.0)	47 (26.6)	5 (10.2)	
Often	20 (8.8)	20 (11.3)	0	
Very frequently	3 (1.3)	3 (1.7)	0	
Q49. How often do you think you should use the internet less?				<0.001
Never	48 (21.2)	24 (13.6)	24 (49.0)	
Rarely	30 (13.3)	22 (12.4)	8 (16.3)	
Sometimes	63 (27.9)	51 (28.8)	12 (24.5)	
Often	54 (23.9)	49 (27.7)	5 (10.2)	
Very frequently	31 (13.7)	31 (17.5)	0	
Q50. How often have you tried to spend less time online but failed?				<0.001
Never	72 (31.9)	41 (23.2)	31 (63.3)	
Rarely	48 (21.2)	36 (20.3)	12 (24.5)	
Sometimes	69 (30.5)	65 (36.7)	4 (8.2)	
Often	28 (12.4)	26 (14.7)	2 (4.1)	
Very frequently	9 (4.0)	9 (5.1)	0	
Q51. How frequently do you prioritize internet use over completing tasks?				<0.001
Never	124 (54.9)	81 (45.8)	43 (87.8)	
Rarely	59 (26.1)	53 (29.9)	6 (12.2)	
Sometimes	27 (11.9)	27 (15.3)	0	
Often	12 (5.3)	12 (6.8)	0	
Very frequently	4 (1.8)	4 (2.3)	0	
Q52. How often do you neglect your responsibilities to go online?				<0.001
Never	100 (44.2)	63 (35.6)	37 (75.5)	
Rarely	73 (32.3)	62 (35.0)	11 (22.4)	
Sometimes	37 (16.4)	36 (20.3)	1 (2.0)	
Often	13 (5.8)	13 (7.3)	0	
Very frequently	3 (1.3)	3 (1.7)	0	
Q53. How often do you go online when you’re feeling down?				<0.001
Never	30 (13.3)	10 (5.6)	20 (40.8)	
Rarely	44 (19.5)	31 (17.5)	13 (26.5)	
Sometimes	63 (27.9)	50 (28.2)	13 (26.5)	
Often	62 (27.4)	59 (33.3)	3 (6.1)	
Very frequently	27 (11.9)	27 (15.3)	0	
Q54. How often do you go online to forget your troubles or negative feelings?				<0.001
Never	44 (19.5)	19 (10.7)	25 (51.0)	
Rarely	48 (21.2)	37 (20.9)	11 (22.4)	
Sometimes	51 (22.6)	40 (22.6)	11 (22.4)	
Often	55 (24.3)	53 (29.9)	2 (4.1)	
Very frequently	28 (12.4)	28 (15.8)	0	
Q55. How often do you get anxious or irritable when you can’t go online?				0.001
Never	108 (47.8)	72 (40.7)	36 (73.5)	
Rarely	72 (31.9)	61 (34.5)	11 (22.4)	
Sometimes	32 (14.2)	30 (16.9)	2 (4.1)	
Often	11 (4.9)	11 (6.2)	0	
Very frequently	3 (1.3)	3 (1.7)	0	
Q56. How often have you felt harassed, threatened, or bullied online?				0.162
Never	174 (77.0)	130 (73.4)	44 (89.8)	
Rarely	35 (15.5)	32 (18.1)	3 (6.1)	
Sometimes	15 (6.6)	13 (7.3)	2 (4.1)	
Often	1 (0.4)	1 (0.6)	0	
Very frequently	1 (0.4)	1 (0.6)	0	

^#^ Fisher’s exact test. Prepared by the authors.

**Table 4:** Descriptive analysis of mental health and psychological well-being in the whole series and segmented by type of responder. The total number of responses is included, with the percentage in brackets.

		Type of Responder		
Question	Whole Series	Student	Staff	*p* Value ^#^
Q57. Have you considered interrupting your studies or taking medical leave for emotional reasons within the past year?				<0.001
No	146 (64.6)	104 (58.8)	42 (85.4)	
Yes	80 (35.4)	73 (41.2)	7 (14.3)	
Q58. Have you been diagnosed with anxiety or depression in the past 12 months?				0.038
No	183 (81.0)	138 (78.0)	45 (91.8)	
Yes	43 (19.0)	39 (22.0)	4 (8.2)	
Q59. Do you think you may have anxiety or depression?				<0.001
No	102 (45.1)	62 (35.0)	40 (81.6)	
Yes (not diagnosed)	80 (35.4)	75 (42.4)	5 (10.2)	
Yes (diagnosed)	44 (19.5)	40 (22.6)	4 (8.2)	
Q60. Have you experienced suicidal ideation in the past year?				0.066
No	189 (83.6)	143 (80.8)	46 (93.9)	
Yes	24 (10.6)	23 (13.0)	1 (2.0)	
Prefer not to answer	13 (5.8)	11 (6.2)	2 (4.1)	
Q61. Have you considered or planned how you might take your own life?				0.034
No	196 (86.7)	148 (83.6)	48 (98.0)	
Yes	22 (9.7)	21 (11.9)	1 (2.0)	
Prefer not to answer	8 (3.5)	8 (4.5)	0	
Q62. In the last 12 months, have you made a suicide attempt?				0.656
No	223 (98.7)	174 (98.3)	49 (100)	
Yes	2 (0.9)	2 (1.1)	0	
Prefer not to answer	1 (0.4)	1 (0.6)	0	
Q63. Have you had a safe space to express these feelings?				<0.001
No	36 (15.9)	34 (19.2)	2 (4.1)	
Yes	103 (45.6)	86 (48.6)	17 (34.7)	
Not needed	87 (38.5)	57 (32.2)	30 (61.2)	
Q64. Are you currently receiving professional help?				<0.001
Yes	50 (22.1)	40 (22.6)	10 (20.4)	
No, but I would like to	55 (24.3)	51 (28.8)	4 (8.2)	
No, I can manage it	46 (20.4)	42 (23.7)	4 (8.2)	
Not needed	75 (33.2)	44 (24.9)	31 (63.3)	
Q65. How often do you see a psychologist or psychiatrist?				0.138
Once a week	3 (2.9)	3 (3.4)	0	
Twice a month	12 (11.8)	9 (10.2)	3 (21.4)	
Once a month	19 (18.6)	16 (18.2)	3 (21.4)	
Less than I would like	13 (12.7)	9 (10.2)	4 (28.6)	
Never	55 (53.9)	51 (58.0)	4 (28.6)	
Q66. Overall, are you satisfied with your life?				0.022
Not at all satisfied	1 (0.4)	1 (0.6)	0	
Somewhat dissatisfied	14 (6.2)	13 (7.3)	1 (2.0)	
Neutral	78 (34.5)	68 (38.4)	10 (20.4)	
Very satisfied	119 (49.6)	82 (46.3)	30 (61.2)	
Completely satisfied	21 (9.3)	13 (7.3)	8 (16.3)	
Q67. Overall level of satisfaction during the past year (on a scale of 0 to 100) *				
Mean ± SD	73 ± 18.1	71.1 ± 18.3	80.0 ± 15.7	0.002 ^a^
Median (range)	80 (0–100)	75 (0–100)	83 (10–100)	<0.001 ^b^

^#^ Chi-square test. ^a^ Student’s *t*-test. ^b^ Mann–Whitney U-test. * The variable “overall satisfaction” was not normally distributed (Kolmogorov–Smirnov test, *p* < 0.001). Prepared by the authors.

**Table 5:** Significantly associated factors for overall dissatisfaction, across the entire series and by respondent group (bivariate logistic regression).

	Whole Series		Students		Staff	
Dissatisfaction ^1^	OR; (95% CI)	*p* Value	OR; (95% CI)	*p* Value	OR; (95% CI)	*p* Value
Age *	0.96 (0.94–0.98)	<0.001		NA ^2^		NA ^2^
Gender						
Male	#Ref cat.					
Female	2.40 (1.25–4.62)	0.009	2.78 (1.18–6.53)	0.019		ns
Subjects from previous years ^3^						
No			#Ref cat.			
Yes		ND	2.52 (1.10–5.82)	0.030		ND
Water pipes						0.013
No	#Ref cat.				#Ref cat.	
Yes	2.79 (1.45–5.38)	0.002		ns	11.1 (2.17–56.9)	0.004
Anxiolytics ^4^						
No	#Ref cat.		#Ref cat.			
Yes	2.31 (1.08–4.92)	0.031	3.46 (1.15–10.4)	0.027		ns
Internet use *	1.08 (1.04–1.11)	<0.001	1.06 (1.01–1.10)	0.008	1.15 (1.04–1.27)	0.006
Excessive internet use ^5^						
No	#Ref cat.		#Ref cat.			
Yes	4.83 (1.66–14.1)	0.004	3.79 (1.26–11.4)	0.018		ND ^6^

Abbreviations: OR, odds ratio; ns, non-significant; ND, non-determined; NA, not applicable; Ref. cat., reference category. * Introduced in the model as a continuous variable. ^1^ The satisfaction cut-off point was set at the 75th percentile of the distribution, with respondents scoring 85 points or below considered dissatisfied. ^2^ Not applicable because age and students/staff are collinear variables. ^3^ This analysis is limited to students only, and therefore cannot be determined in the whole series and staff. ^4^ It includes anxiolytics, sedatives, and/or hypnotics. ^5^ A cut-off score of 40 was determined based on the 75th percentile of the total score on the 15-item Likert scale (range 15–75). Individuals scoring above this threshold were categorized as excessive internet users. ^6^ None of the staff exceeded the 75th percentile in terms of excessive internet use score. Prepared by the authors.

## Data Availability

The raw data supporting the conclusions of this article will be made available by the authors on request.

## Supplementary Materials

The following supporting information can be downloaded at: https://www.mdpi.com/article/10.3390/healthcare13080918/s1, Figure S1: Box plot representing the difference in overall satisfaction score by gender (A), consumption of water pipes (B), consumption of anxiolytics (C), and excessive internet use (D), defined as responders who scored above the percentile 75th (40 points) in the aggregated internet use score. The lines represent the median, the box edges represent the 25th and 75th percentiles of the distribution, and the whiskers represent the range. Overall satisfaction did not follow a normal distribution (Kolmogorov–Smirnov test, *p* < 0.001), thus the Mann–Whitney U-test was used for the calculation of *p* values. The dashed lines mark the 75th percentile of the overall satisfaction (cut off = 85 points). Table S1: Descriptive analysis of the consumption of smoked tobacco, water pipes, and electronic nicotine delivery systems (ENDSs) in the whole series and segmented by type of responder; Table S2: Significantly associated factors for tobacco consumption, ENDS consumption, water pipe use, anxiolytic consumption, illicit drug use, and excessive internet use (bivariate logistic regression); Table S3: Descriptive analysis of the consumption of psychotropic drugs (anxiolytics, sedatives, and/or hypnotics) in the whole series and segmented by type of responder; Table S4: Descriptive analysis of the consumption of drugs of abuse in the whole series and segmented by type of responder; Table S5: Descriptive analysis of mental health and psychological well-being segmented by gender. All supplementary figures and tables were prepared by the authors.
