# Supplementary material for: Psychological Well-Being, Substance Use, and Internet Consumption Among Students and Teaching Staff of the Faculty of Veterinary Medicine: Risk and Protective Factors Associated with Well-Being and Dissatisfaction
Source: Healthcare (Basel). 2025 Apr 16;13(8):918. doi: 10.3390/healthcare13080918 (PMC12026891; doi:10.3390/healthcare13080918)
Supplement: Supplementary file 1 [file healthcare-13-00918-s001.zip › Table S1.pdf]

Table S1. Descriptive analysis of the consumption of smoked tobacco, water pipes and electronic nicotine delivery systems (ENDS) in the whole series and segmented by type of responder. The total number of responses is included, with the percentage in brackets.

Question	Whole series	Type of responder		P value[#]
		Students	Staff	
Q16. Have you smoked cigarettes or other tobacco products?				
Never	105 (46.5)	86 (48.6)	19 (38.8)	0.149
Sometime in life	80 (35.4)	61 (34.5)	19 (38.8)	
In the last 12 months	11 (4.9)	10 (5.6)	1 (2.0)	
In the last 30 days	11 (4.9)	9 (5.1)	2 (4.1)	
Daily	19 (8.4)	11 (6.2)	8 (16.3)	
*Q17. In the past 30 days, what is your average daily cigarette count?**				
Median (Range)	105.5 (1 – 600)	45 (1 – 600)	180 (1 – 300)	0.121[a]
*Q18. In the last 30 days, what kind of cigarettes have you smoked?**				0.808
Pre-rolled cigarettes	14 (41.2)	9 (39.1)	5 (45.5)	
Hand-rolled cigarettes	15 (44.1)	10 (43.5)	4 (45.5)	
Both	5 (14.7)	4 (17.4)	1 (9.1)	
*Q19. At what age did you initiate daily tobacco use? (years)**				
Mean ± SD	18.8 ± 4.8	17.8 ± 2.6	20.3 ± 6.6	0.077[b]
Median (range)	18 (14 – 42)	17 (14 – 24)	20 (14 – 42)	0.263[a]
*Q20. Have you ever considered quitting smoking?**				0.201
No	3 (9.4)	1 (4.8)	2 (18.2)	
Yes, and I have tried	21 (65.6)	13 (61.9)	8 (72.7)	
Yes, but I have not made an attempt	8 (25.0)	7 (33.3)	1 (9.1)	
Q21. Have you used water pipes or hookahs?				0.042
Never	107 (47.3)	77 (43.5)	30 (61.2)	
Sometime in life	100 (44.2)	81 (45.8)	19 (38.8)	
In the last 12 months	13 (5.8)	13 (7.3)	0	
In the last 30 days	6 (2.7)	6 (3.4)	0	
Daily	0	0	0	
*Q22. Number of uses in the past 12 months.***				0.970
Median (range)	2 (1 – 24)	2 (1 – 24)	3 (1 – 5)	
Q23. Have you ever used e-cigarettes?				<0.001
Never	123 (54.4)	81 (45.8)	42 (85.7)	
Sometime in life	71 (31.4)	66 (37.3)	5 (10.2)	
In the last 12 months	13 (5.8)	13 (7.3)	0	
In the last 30 days	15 (6.6)	15 (8.5)	2 (4.1)	
Daily	4 (1.8)	2 (1.1)	0	
*Q24. At what age did you start vaping regularly? (years)****				
Mean ± SD	27.2 ± 10.7	23.3 ± 7.1	44 ± 5.6	<0.001[b]
Median (range)	22 (18 – 52)	20 (18 – 41)	42.5 (39 – 52)	0.004[a]
*Q25. What type of cartridges do you use?****				0.014
Ceased consumption	73 (70.9)	70 (72.9)	3 (42.9)	
With nicotine	18 (17.5)	14 (14.6)	4 (57.1)	
Without nicotine	12 (11.7)	12 (12.5)	0	

[#]Chi square test.
*Only among responders who have smoked.
**Only among responders who have used water pipes in the last 12 months.
***Only among responders who have used ENDS.
[a]Mann-Whitney U-test.
[b]Student t-test.
Prepared by the authors.
