# Supplementary material for: Psychological Well-Being, Substance Use, and Internet Consumption Among Students and Teaching Staff of the Faculty of Veterinary Medicine: Risk and Protective Factors Associated with Well-Being and Dissatisfaction
Source: Healthcare (Basel). 2025 Apr 16;13(8):918. doi: 10.3390/healthcare13080918 (PMC12026891; doi:10.3390/healthcare13080918)
Supplement: Supplementary file 1 [file healthcare-13-00918-s001.zip › Table S2.pdf]

**Table S2.** Significantly associated factors for tobacco consumption, ENDS consumption, water pipe use, anxiolytic consumptions, illicit drug use and excessive internet use (bivariate logistic regression).

| | OR; (95% CI) | P value |
|---|---|---|
| Tobacco consumption | | |
| Age* | 1.03 (1.01 – 1.03) | 0.009 |
| ENDS consumption | | |
| Age* | 0.95 (0.92- 0.98) | <0.001 |
| Water pipe use | | |
| Students | #Ref cat. | |
| Teaching staff | 6.09 (1.46 – 25.4) | 0.013 |
| Anxiolytic consumption | | |
| Age* | 1.03 (1.01 – 1.05) | 0.012 |
| Ilicitit brug use | | |
| Age* | 1.03 (1.01 – 1.05) | 0.012 |
| Excessive internet use** | | |
| Age* | 0.91 (0.85 – 0.96) | 0.001 |
| Male | #Ref cat. | |
| Female | 2.42 (1.11 – 5.30) | 0.027 |
| Energy drink consumption (yes) | 2.19 (1.18 – 4.06) | 0.013 |
| Water pipe use (yes) | 3.11 (1.60 – 6.04) | 0.001 |
| ENDS consumption (yes) | 2.65 (1.41 – 4.97) | 0.002 |

Abbreviations: OR, odds ratio; Ref. cat., reference category.

* Introduced in the model as a continuous variable.

**A cut-off score of 40 was determined based on the 75$^{th}$ percentile of the total score on the 15-item Likert scale (range 15 – 75). Individuals scoring above this threshold were categorized as excessive internet users.

Prepared by the authors.
