# Supplementary material for: Psychological Well-Being, Substance Use, and Internet Consumption Among Students and Teaching Staff of the Faculty of Veterinary Medicine: Risk and Protective Factors Associated with Well-Being and Dissatisfaction
Source: Healthcare (Basel). 2025 Apr 16;13(8):918. doi: 10.3390/healthcare13080918 (PMC12026891; doi:10.3390/healthcare13080918)
Supplement: Supplementary file 1 [file healthcare-13-00918-s001.zip › Table S3.pdf]

Table S3. Descriptive analysis of the consumption of psychotropic drugs (anxiolytics, sedatives, and/or hypnotics) in the whole series and segmented by type of responder. The total number of responses is included, with the percentage in brackets.

		Type of responder		
Question	Whole series	Students	Staff	P value[#]
Q26. Have you used any anxiolytics, sedatives, or hypnotics?				0.193
Never	153 (67.7)	125 (70.6)	28 (57.1)	
Sometime in life	52 (23.0)	35 (19.8)	17 (34.7)	
In the last 12 months	12 (5.3)	10 (5.6)	2 (4.1)	
In the last 30 days	7 (3.1)	6 (3.4)	1 (2.0)	
Daily	2 (0.9)	1 (0.6)	1 (2.0)	
*Q27. At what age did you initiate the use? (years)**				
Mean ± SD	23.6 ± 8.8	19.6 ± 3.2	33.9 ± 10.5	<0.001[a]
Median (Range)	20 (15 – 59)	19 (15 – 30)	30 (22 – 59)	<0.001[b]
*Q28. When was the first time you consumed it?**				0.596
≤ 1 year	10 (13.7)	7 (13.5)	3 (14.3)	
> 1 year	63 (86.3)	45 (86.5)	18 (85.7)	
*Q29. What is your primary source for obtaining it?**				0.666
Own prescription	46 (63.0)	34 (65.4)	12 (57.1)	
Prescription for other	19 (26.0)	12 (23.1)	7 (33.3)	
Internet	0	0	0	
From unknown person	0	0	0	
Over the counter	8 (11.0)	6 (11.5)	2 (9.5)	
*Q30. In the past 12 months, I have combined these medications with…**				0.199
I have not combined	52 (96.3)	39 (97.5)	13 (92.9)	
Alcohol	1 (1.4)	1 (2.5)	0	
Illicit substances	1 (1.4)	0	1 (7.1)	
Both	12 (11.7)	12 (12.5)	0	

Abbreviations: SD, standard deviation.

[#]Chi square test.

*Only among responders who have used psychotropic drugs.

[a]Student t-test.

[b]Mann-Whitney U-test.

Prepared by the authors.
