# Supplementary material for: Psychological Well-Being, Substance Use, and Internet Consumption Among Students and Teaching Staff of the Faculty of Veterinary Medicine: Risk and Protective Factors Associated with Well-Being and Dissatisfaction
Source: Healthcare (Basel). 2025 Apr 16;13(8):918. doi: 10.3390/healthcare13080918 (PMC12026891; doi:10.3390/healthcare13080918)
Supplement: Supplementary file 1 [file healthcare-13-00918-s001.zip › Table S4.pdf]

Table S4. Descriptive analysis of the consumption of drugs of abuse in the whole series and segmented by type of responder. The total number of responses is included, with the percentage in brackets.

Question	Whole series	Type of responder		P value[#]
		Students	Staff	
Q31. Have you ever consumed cannabis, marihuana or hashish?				0.235
Never	124 (54.9)	103 (58.2)	21 (42.9)	
Sometime in life	77 (34.1)	55 (31.1)	22 (44.9)	
In the last 12 months	15 (6.6)	12 (6.8)	3 (6.1)	
In the last 30 days	8 (3.5)	5 (2.8)	3 (6.1)	
Daily	2 (0.9)	2 (1.1)	0	
*Q32. At what age did you first use cannabis? (years)**				
Mean ± SD	18.4 ± 3.2	18.1 ± 3.1	19.4 ± 3.5	0.063[a]
Median (Range)	17.5 (12 – 30)	17 (12 – 30)	18.5 (15 – 26)	0.110[b]
*Q33. When was the first time you consumed it?**				0.184
≤ 1 year	6 (5.9)	6 (8.1)	0	
> 1 year	96 (94.1)	68 (91.9)	28 (100)	
Q34. Have you ever consumed cocaine?				0.001
Never	208 (92.0)	169 (95.5)	39 (79.6)	
Sometime in life	15 (6.6)	6 (3.4)	9 (18.4)	
In the last 12 months	3 (1.3)	2 (1.1)	1 (2.0)	
In the last 30 days	0	0	0	
Daily	0	0	0	
*Q35. When was the first time you consumed cocaine?***				0.556
≤ 1 year	1 (5.6)	0	1 (10.0)	
> 1 year	17 (94.4)	8 (100)	9 (90.0)	
Q36. Have you ever consumed MDMA?				0.355
Never	208 (92.0)	165 (93.2)	43 (87.8)	
Sometime in life	9 (4.0)	6 (3.4)	3 (6.1)	
In the last 12 months	7 (3.1)	4 (2.3)	3 (6.1)	
In the last 30 days	2 (0.9)	2 (1.1)	0	
Daily	0	0	0	
*Q37. When was the first time you consumed MDMA?****				0.529
≤ 1 year	2 (11.1)	2 (16.7)	0	
> 1 year	16 (88.9)	10 (83.3)	6 (100)	
Q38. Have you ever consumed amphetamines?				0.001
Never	209 (92.5)	170 (96.0)	39 (79.6)	
Sometime in life	15 (6.6)	6 (3.4)	9 (18.4)	
In the last 12 months	2 (0.9)	1 (0.6)	1 (2.0)	
In the last 30 days	0	0	0	
Daily	0	0	0	
Q39. When was the first time you consumed amphetamines?†				0.412
≤ 1 year	1 (5.9)	1 (14.3)	0	
> 1 year	16 (94.1)	6 (85.7)	10 (100)	
Q40. Have you ever consumed volatile inhalants (poppers)?				0.330
Never	203 (89.8)	158 (89.3)	45 (91.8)	
Sometime in life	13 (5.8)	9 (5.1)	4 (8.2)	
In the last 12 months	7 (3.1)	7 (4.0)	0	
In the last 30 days	3 (1.3)	3 (1.7)	0	
Daily	0	0	0	
Q41. When was the first time you consumed volatile inhalants (poppers)?† †				0.654
≤ 1 year	5 (21.7)	4 (21.1)	1 (25.0)	
> 1 year	18 (78.3)	15 (78.9)	3 (75.0)	

Abbreviations: SD, standard deviation.

[#]Chi square test.

*Only among responders who have consumed cannabis, marihuana or hashish.

** Only among responders who have consumed cocaine.

***Only among responders who have consumed MDMA.
†Only among responders who have consumed amphetamines.
††Only among responders who have consumed volatile inhalants (poppers).
[a]Student t-test.
[b]Mann-Whitney U-test.
Prepared by the authors.
