# Supplementary material for: Psychological Well-Being, Substance Use, and Internet Consumption Among Students and Teaching Staff of the Faculty of Veterinary Medicine: Risk and Protective Factors Associated with Well-Being and Dissatisfaction
Source: Healthcare (Basel). 2025 Apr 16;13(8):918. doi: 10.3390/healthcare13080918 (PMC12026891; doi:10.3390/healthcare13080918)
Supplement: Supplementary file 1 [file healthcare-13-00918-s001.zip › Table S5.pdf]

Table S5. Descriptive analysis of mental health and psychological well-being segmented by gender. The total number of responses is included, with the percentage in brackets.

Question	Female (n = 162)	Male (n = 64)	P value[#]
Q57. Have you considered interrupting your studies or taking medical leave for emotional reasons within the past year?			
No	98 (60.5)	48 (75.0)	0.040
Yes	64 (39.5)	16 (25.0)	
Q58. Have you been diagnosed with anxiety or depression in the past 12 months?			0.232
No	128 (79.0)	55 (85.9)	
Yes	34 (21.0)	9 (14.1)	
Q59. Do you think you may have anxiety or depression?			<0.001
No	59 (36.4)	43 (67.2)	
Yes (not diagnosed)	66 (40.7)	14 (21.9)	
Yes (diagnosed)	37 (22.8)	7 (10.9)	
Q60. Have you experienced suicidal ideation in the past year?			0.142
No	131 (80.9)	58 (90.6)	
Yes	19 (11.7)	5 (7.8)	
Prefer not to answer	12 (7.4)	1 (1.6)	
Q61. Have you considered or planned how you might take your own life?			0.159
No	137 (84.6)	59 (92.2)	
Yes	17 (10.5)	5 (7.8)	
Prefer not to answer	8 (4.9)	0	
Q62. In the last 12 months, have you made a suicide attempt?			0.634
No	160 (98.8)	63 (98.4)	
Yes	1 (0.6)	1 (1.6)	
Prefer not to answer	1 (0.6)	0	
Q63. Have you had a safe space to express these feelings?			0.018
No	28 (17.3)	8 (12.5)	
Yes	81 (50.0)	22 (34.4)	
Not needed	53 (32.7)	34 (53.1)	
Q64. Are you currently receiving professional help?			0.001
Yes	39 (24.1)	11 (17.2)	
No, but I would like to	44 (27.2)	11 (17.2)	
No, I can manage it	38 (23.5)	8 (12.5)	
Not needed	41 (25.3)	34 (53.1)	
Q65. How often do you see a psychologist or psychiatrist?			0.824
Once a week	3 (3.8)	0	
Twice a month	10 (12.5)	2 (9.1)	
Once a month	14 (17.5)	5 (22.7)	
Less than I would like	9 (11.3)	4 (18.2)	
Never	44 (55.0)	11 (50.0)	
Q66.Overall, are you satisfied with your life?			0.126
Not at all satisfied	1 (0.6)	0	
Somewhat dissatisfied	12 (7.4)	2 (3.1)	
Neutral	62 (38.3)	16 (25.0)	
Very satisfied	74 (45.7)	38 (59.4)	
Completely satisfied	13 (8.0)	8 (12.5)	
*Q67. Overall level of satisfaction during the past year (on a scale of 0 to 100)**			
Mean ± SD	70.6 ± 18.9	79.0 ± 14.1	0.002[a]
Median (range)	75 (0 – 100)	80 (25 – 100)	0.001[b]

[#]Chi square test.

[a]Student T-test.

[b]Mann Whitney U-test.

*The variable “overall satisfaction” was not normally distributed (Kolmogorov-Smirnov test, P < 0.001). Prepared by the authors.
