# Supplementary figures and images for: Psychological Well-Being, Substance Use, and Internet Consumption Among Students and Teaching Staff of the Faculty of Veterinary Medicine: Risk and Protective Factors Associated with Well-Being and Dissatisfaction
Source: Healthcare (Basel). 2025 Apr 16;13(8):918. doi: 10.3390/healthcare13080918 (PMC12026891; doi:10.3390/healthcare13080918)

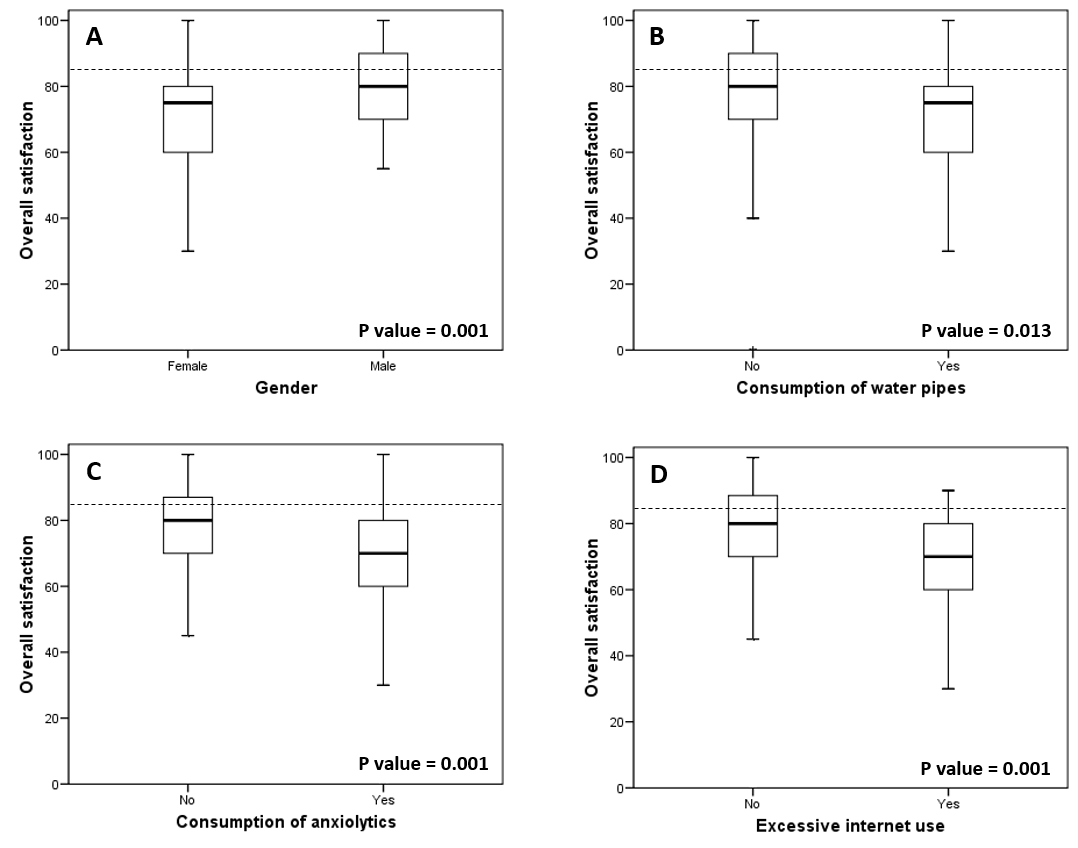

Supplement: Supplementary file 1 [file healthcare-13-00918-s001.zip › Figure S1.tif]
